# A retrospective observational study on case reports of adverse drug reactions (ADRs) to tirzepatide

**DOI:** 10.3389/fphar.2025.1608657

**Published:** 2025-07-01

**Authors:** Mengmeng Huang, Guangwei Liu, Chuanzhou Zhang, Ying Wang, Shengfen Liu, Jun Zhao

**Affiliations:** ^1^ Department of Pharmacy, The Affiliated Hospital of Qingdao University, Qingdao, Shandong, China; ^2^ Department of Pharmacy, Lu 'an Hospital of Anhui Medical University, Lu 'an, Anhui, China; ^3^ Department of Gastrointestinal Surgery, The Affiliated Hospital of Qingdao University, Qingdao, Shandong, China; ^4^ Department of Pharmacy, Mengyin County People’s Hospital, Linyi, Shandong, China; ^5^ Department of Pharmacy, The Third People’s Hospital of Huangdao District, Qingdao, Shandong, China

**Keywords:** ADR, case report, systematic analysis, tirzepatide, adverse drug reactions

## Abstract

**Background:**

With the increasing clinical use of tirzepatide, its safety profile has garnered significant attention. This article systematically reviews case reports of tirzepatide-associated adverse drug reactions (ADRs) to summarize their characteristics.

**Method:**

We searched PubMed, Web of Science, ScienceDirect, Wiley Online, and Embase databases for case reports on tirzepatide adverse events using the keywords: “tirzepatide”, “adverse reaction”, “adverse event”, “side effect”, “safety”, “case report”, “induced”, “associated”, and “related”. Statistical analysis was performed on the identified cases.

**Results:**

A total of 43 cases of tirzepatide ADR were identified from 37 articles. Among these patients (24 female, 19 male; mean age 50.23 ± 17.24 years), 19 involved concomitant medications affecting multiple systems. ADR was reported in each dosage of tirzepatide, with the most occurring at 2.5–5 mg (16 cases), and primarily occurred within 1–6 months of initiation. Regarding rechallenge, 15 patients discontinued tirzepatide, three continued use, and one reduced the dose. ADR involved seven gastrointestinal tract and endocrine systems, including liver and gallbladder, circulation, nerve, skin, and urinary. Notable manifestations included ketoacidosis, liver injury, hypotension, intestinal obstruction, and allergic reactions. Among them, ketoacidosis and common peroneal neuropathy causing foot sagging, acute appendicitis, lower limb venous thrombosis, gastric outlet obstruction, gastric emptying delay, and acute liver injury were not mentioned in the drug instructions. ADR correlation assessment was performed for 8 patients:4 cases of cardiovascular events and ketoacidosis were all evaluated as “probable” using the Naranjo scale, 3 cases of liver injury were assessed by RUCAM (2 case as “possible”, 1 cases as “probable”), 1 case did not specify the evaluation method, with the result being “highly probable”. All 43 patients underwent ADR correlation re-evaluation:32 cases (74.42%) were assessed as “probable”,11 cases (25.58%) were assessed as “possible”.

**Conclusion:**

Tirzepatide-associated ADRs most commonly occur within the first 6 months of treatment, primarily affecting the digestive, endocrine, liver, and gallbladder systems. Enhanced monitoring of liver and kidney function is warranted, especially in patients concurrently taking other potentially hepatotoxic or nephrotoxic medications. Additionally, intensified therapeutic drug monitoring is recommended for patients with cardiovascular disease, those requiring weight-based dosing adjustments, and those experiencing rapid weight loss.

## 1 Introduction

Tirzepatide is the first dual-target agonist that targets both the glucagon-like peptide-1 receptor (GLP-1R) and the glucose-dependent insulinotropic polypeptide (GIP) receptors, resulting in significant improvements in glucose control and weight without increasing the risk of hypoglycemia (Rosenstock et al., 2021). Approved in the United States in May 2022 by the Food and Drug Administration (FDA) for glycemic control in adults with type 2 diabetes, tirzepatide received weight loss approval in November 2023. The FDA approved tirzepatide in December 2024 as the first drug to treat adults with moderate to severe obstructive sleep apnea (OSA), marking a significant milestone in OSA treatment (FDA, 2024). It is currently in Phase III clinical trials for the treatment of heart failure, obesity, and cardiovascular diseases associated with type 2 diabetes, along with Phase II trials for non-alcoholic steatohepatitis (Syed, 2022). As can be seen, tirzepatide has a substantial clinical application potential.

As tirzepatide is increasingly used in type 2 diabetes, obesity, OSA, and other fields, adverse effects such as injection site reactions and gastrointestinal symptoms, warrant increased attention (Caruso et al., 2024). There is currently no literature to assess its safety. Therefore, this study systematically reviewstirzepatide-related ADR case reports to analyzeoccurrence pattern, causes, severity, preventability, and outcomes, providing a foundation for safe and effective use.

## 2 Methods

### 2.1 Source and search strategy

The search for the terms “tirzepatide”, “adverse reaction”, “adverse event”, “side effect”, “safety”, “case report”, “induced”, “associated”, and “related” was conducted in PubMed, Web of Science, ScienceDirect, Wiley Online, and Embase databases. The search time was from the inception of the library until March 2025. The search was performed using subject headings alongside free words. Two researchers independentlyscreened articles according to the inclusion and exclusion criteria. Differences were resolved through consensus or consultation with a third reviewer, with the literature being decided to be included as part of the discussion.

### 2.2 Qualification criteria and research

The inclusion criteria for this study were: (1) Publicly published clinical case reports (2) complete clinical data, such as patient information (gender, age), primary disease, ADR occurrence and outcome. Exclusion criteria included: (1) non-case reporting literature or non-English studies, such as reviews, experimental research, observational research, and other non-English studies; (2) incomplete literature that could not be analyzed; and (3) duplicate publications.

### 2.3 Data extraction and quality assessment

This study collected data on the year, country, age, gender, primary disease, dose during ADR occurrence, time to first onset, clinical manifestations, intervention measures, drug re-challenge, outcome and outcome time, combined medication use, and ADR correlation evaluation. The ADRs in the included cases were re-evaluated for causality using the Naranjo Adverse Drug Reaction Probability Scale. The Naranjo assessment method primarily summarizes the scores based on Naranjo’s ten detailed rules, dividing the total score results into four levels: “definite”,“probable”,“possible”,“doubtful”. Data was entered into Microsoft Excel worksheet for statistics.

### 2.4 Statistical analysis

Statistical analysis was performed using SPSS (version 29.0.1.0). Continuous variables were expressed as mean ± standard deviation, while categorical variables were expressed as frequency and percentage.

## 3 Results

### 3.1 Search results and case characteristics

**FIGURE 1:**
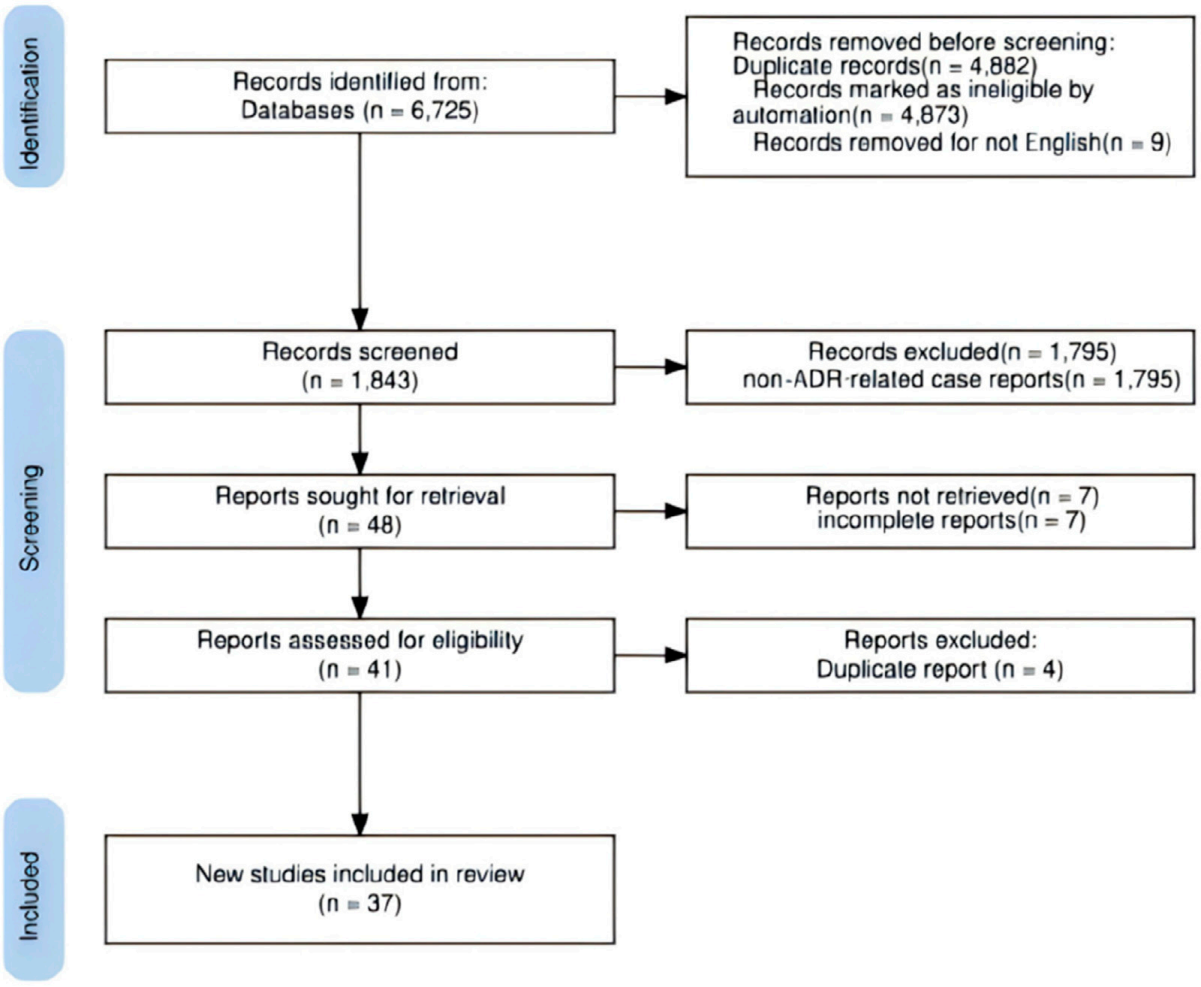
Document screening flow chart (PROSPERO).

According to the inclusion and exclusion criteria, 37 articles meeting the criteria were included (Chan et al., 2025; Morgenthaler and Depietro, 2024; Mathew and Hannoodee, 2023; Rao and Nimako, 2023; Peicher et al., 2023; Bayless et al., 2024; Betchvaia et al., 2024; Mando et al., 2024; Gordon et al., 2023; Grennan et al., 2024; Gomez Casanovas et al., 2023; Weber et al., 2023; Iskander et al., 2025; Fadel et al., 2024; Abdullah et al., 2024; Klein et al., 2024; Sohal et al., 2024; Fontana et al., 2024; Terrington et al., 2024; Iqbal et al., 2024; Louwagie et al., 2025; Bitar et al., 2024; Gurel et al., 2024; Mercer et al., 2023a; Karakus et al., 2024; Mercer et al., 2023b; Le et al., 2024; He et al., 2023; Mizumoto, 2023; Tucker and Ritchie, 2024; Jamail et al., 2024; Bobbs and Howland, 2024; Rivera et al., 2023; Kido et al., 2024; Farooqi et al., 2024; Aleman et al., 2023; Fisher et al., 2025), reporting 43 cases. Figure 1 illustrates the literature screening process. The reported ADR cases originated most frequently from the United States (33/43), followed by Kuwait (6/43), the United Kingdom (1/43), Japan (1/43), the United Arab Emirates (1/43), and Nigeria (1/43). The 43 patients ranged in age from 17 to 77 years, averaging 50.23 ± 17.24 years. There were 24 female cases (mean age 42.96 ± 16.43 years) and 19 male cases (mean age 59.42 ± 13.45 years). Body mass index (BMI) data were available for 20 patients, averaging 33.02 ± 5.69 kg/m^2^. Detailed case characteristics are provided in Supplementary Table S1.

### 3.2 Purpose of medication and dosage

Among the 43 patients, dosage information was not reported for 16 cases, and the primary disease was not documented in one case. Table 1 contains information about the ADR for tirzepatide, including the dose and purpose.

**TABLE 1:** Dosage and purpose of tirzepatide when ADR occurred.

Dose	T2DM, obesity	T2DM	Obesity	Overweight	Not mentioned	T1DM, obesity	Pre-diabetes	Total
2.5 mg	3	2	1	2				8
5 mg		2	5					7
7.5 mg		2	3				1	6
10 mg					1	1		2
12 mg			1					1
12.5 mg	1		1					2
15 mg		1						1
Not mentioned	5	3	5	1	2			16
Total	9	10	16	3	3	1	1	43

### 3.3 ADR occurrence time, outcome, and re-challenge

Of the 43 patients, ten did not specify the duration of medication when the ADR occurred (including two cases occurred during the first use), two cases (intestinal obstruction and allergies) occurred within 30 min, six occurred within 1 week, five occurred within 4 weeks, 16 occurred between January and June, and four occurred between June and December.

41 patients improved or recovered after discontinuing the drug and receiving symptomatic treatment, one died, and one had a poor prognosis. Outcome timing: Thirty patients improved after treatment but did not clearly record whether the medication was stopped during treatment, seven improved after treatment, three improved after treatment, two did not improve after treatment, and one did not stop the medication (dose reduction of the combination drug).

21 patients did not specify the outcome time, 9 patients within 1 week, 11 patients within one to 4 weeks, and 2 patients within 6 weeks. Drug rechallenge situation: 25 patients did not mentioned whether they should continue using it, 15 patientsdiscontinued the durg (including one did not stop using it at first but had recurring symptoms since), 3 patients continued using it (with one reduced the dose).

### 3.4 ADR involvement system and clinical manifestations

ADRs in 43 patients affected seven organ systems, including the gastrointestinal tract, endocrine, hepatobiliary, and circulatory. Clinical manifestations included intestinal obstruction, ischemia, ketoacidosis, liver injury, hypotension, and allergies. Table 2 details the information ofthe ADR involvement system and clinical manifestations.

**TABLE 2:** ADR involvement system and clinical manifestations.

System	Number of cases (% of the proportion)	Main manifestations (number of examples)
Gastrointestinal tract	14 (32.56)	Intestinal obstruction (4) Pancreatitis (3) Intestinal ischemia (2) Acute appendicitis (1) Gastric outlet obstruction (1) Delayed gastric emptying (1) Pancreatic cancer (1) Gastric dilation gastric stones (1)
Endocrine	10 (23.26)	Hypoglycemia ketoacidosis (non-diabetic patients) (4) Norm-glycemia ketoacidosis (non-diabetic patients) (1) Norm-glycemia ketoacidosis (diabetic patients) (2) Ketoacidosis (non-diabetic patients) (2) Rapid weight loss-related thyrotoxicosis (1)
Liver and gallbladder	5 (11.63)	Acute liver failure (1) Acute liver injury, coagulation dysfunction (1) Liver injury (1) Liver injury, increased drug concentration (1) Jaundice (1)
Nerve	5 (11.63)	Peroneal neuropathy causes foot sagging (2) Migraine (1) Paranoid and hallucination (1) Ataxia, encephalopathy (1)
Cycle	4 (9.30)	Hypotension, tachycardia (2) Hypotension (1) Extension in the lower limb vein (1)
Skin	4 (9.30)	Allergies (Binophase allergic reaction) (1) Systemic allergies (1) Rash and pain in the injection site (1) Pigmented Liver Planus (1)
Urination	1 (2.33)	Acute renal injury (1)

### 3.5 ADR correlation evaluation

Of the 43 patients, ADR correlation assessment was performed for 8 patients using either the Naranjo or RUCAM methods. RUCAM is a standardized scoring system designed to evaluate the likelihood of a causal link between drug-induced liver injury (DILI) and the implicated drug. The score classifies ADR’s causal relationship as excluded, unlikely, possible, probable, or highly probable. The results showed that 4 cases of cardiovascular events and ketoacidosis were all evaluated as “probable” using the Naranjo scale, 3 cases of liver injury were assessed by RUCAM (2 case as “possible”, 1 cases as “probable”), 1 case did not specify the evaluation method, with the result being “highly probable”. The causal relationship of adverse reactions was re-evaluated in 43 patients: 32 cases (74.42%) were evaluated as “probable”, and 11 cases (25.58%) were evaluated as “possible”. The evaluation results of 6cases in the original literature were consistent with the re-evaluation results.1 case was evaluated as “highly probable” in the original literature and “probable” in the re-evaluation, 1 case was evaluated as “possible” in the original literature and “probable” in the re-evaluation. The detailed evaluation results are shown in Supplementary Table S1.

## 4 Discussions

Tirzepatide is the first dual-target agonist that acts on GLP-1 and GIP receptors, significantly improving glycemic control and weight without increasing the risk of hypoglycemia (Rosenstock et al., 2021). Current research indicates that in patients with type 2 diabetes, tirzepatide outperforms other GLP-1 receptor agonists, such as semaglutide, regarding glycosylated hemoglobin and weight control (Frías et al., 2021).

### 4.1 ADR features

In this study, the male-to-female ratio of patients was 1.26:1, with adverse drug reactions occurring in all age groups. Due to the small number of statistical cases, the relationship between gender, age, and tirzepatide ADR cannot be established. The patient’s original condition was diabetes, obesity, or both. Three overweight patients did not indicate whether they had weight-related comorbidities, so their medications were taken as prescribed. Antihypertensive drugs, fat-adjusting drugs, contraceptives, antidepressants, thyroid hormone supplements, antidiabetic drugs, anticoagulants, and other medications were prescribed to 19 of the 43 patients. Due to the patients’ complex medication situation, no correlation was found between ADR occurrence and the original disease or combination of medications. Of the 43 patients, nine did not mention whether the dose increase was performed as required, eight increased according to the recommended dose, nine were treated at 2.5 mg per week, and one (ADR is acute liver failure) clearly stated (Terrington et al., 2024) that the patient did not increase at 2.5 mg when increasing the dose. This variation could be related to the occurrence of ADR. The relationships between ADRs, dose size, and increased standardization remain unknown.

### 4.2 ADR generation mechanism

#### 4.2.1 Gastrointestinal ADR

Appendicitis, intestinal obstruction, colon ischemia, delayed gastric emptying, gastric outlet obstruction, pancreatitis, and pancreatic cancer are examples of gastrointestinal adverse drug reactions. Tirzepatide decreases gastric motility by inhibiting gastrointestinal motility and increasing pyloric region contraction, potentility causing fecal stasis, appendix obstruction, increased intraluminal pressure, inflammation, bacterial overgrowth, infection, and appendiceal mucositis (Chan et al., 2025). The GLP-1R is expressed in normal pancreatic tissues. GLP-1RA directly increases pancreatic enzyme secretion through GLP-1R expressed by pancreatic acini cells. In some patients treated with GLP-1RA, the levels of circulating pancreatic enzymes increase, thereby having an important impact on the pancreas (Drucker, 2024). Naranjo evaluated one case of adverse reaction to pancreatic cancer as “possible”, but with a score of only 2. 90% of pancreatic cancers occur in exocrine regions, and their development may take several years. The impact of this drug category on the occurrence of pancreatic cancer is still unclear, and more clinical evidence is needed for support (Betchvaia et al., 2024).

The instructions for tirzepatide currently do not mention adverse events of intestinal obstruction; however, in September 2023, the FDA added a warning about intestinal obstruction to the label of tirzepatide (U.S. Food and Drug Administration, 2023), emphasizing the importance of raising awareness about the potential side effects and severity of tirzepatide, particularly for patients with a history of colon surgery, multiple abdominal surgeries, or intestinal obstruction. Colon ischemia may be associated with decreased oral intake caused by glucagon-like peptide-1 receptor agonists (GLP-1RAs), which can result in systemic hypotension and, ultimately, ischemic colitis. Patients with a history of colon ischemia attacks, peripheral vascular disease, congestive heart failure, or irritable bowel syndrome, for example, should drink plenty of water while taking GLP-1RA to lower their risk, assuming no contraindications (Bayless et al., 2024). In this study, a patient with tirzepatide was delayed due to delayed gastric emptying, resulting in gastrointestinal tract clearance during the procedure. In a retrospective study of patients who underwent esophageal gastroduodenoscopy (EGD), Nadeem et al. discovered that the risk of GLP-1RA and gastric contents retention was four times higher, as was the risk of EGD termination (Nadeem et al., 2024). Patients taking drugs containing the GLP-1RA component may experience slower intestine clearance during the perioperative period.

#### 4.2.2 ADR of the endocrine system

Endocrine ADR is primarily ketoacidosis, which includes normal blood sugar acidosis, and it affects both diabetic and non-diabetic patients. Starvation ketoacidosis is a potential cause of diabetic ketoacidosis (DKA) in obese patients who restrict their daily calorie intake to less than 500 calories (Cahill, 2006). Mild ketosis typically begins after fasting for 12–14 h, but it can progress to ketoacidosis in severe nutritional deficiency, ketoacidosis, pregnancy, and other conditions that necessitate hospitalization (Slade and Ashurst, 2020). Second, it could be linked to insulin resistance and metabolic abnormal obesity (Samocha-Bonet et al., 2014), and insulin resistance may indicate relative insulin deficiency. Insulin withdrawal or dose reduction is important in inducing DKA during GLP-1RA treatment (Yang et al., 2022). Euglycemic diabetic ketoacidosis (EDKA) is a subtype of DKA. Patients exhibit DKA symptoms, but their blood sugar levels are less than 250 mg/dL (typically less than 100 mg/dL) (Rawla et al., 2017; Plewa et al., 2025). Among the 9 patients with ketoacidosis, 2 patients, although combined with SGLT2 inhibitors, had been taking the SGLT2 inhibitors as planned. The symptoms of ketoacidosis occurred respectively after the replacement of tirzepatideand the injection of tirzepatide. The adverse reaction symptoms showed a temporal association with tirzepatide administration. It was considered that ketoacidosis was caused by tirzepatide. In two previous studies, there was no ketoacidosis in 405 patients who took SGLT-2i and tirzepatide. More research was needed to determine the absolute risk of EDKA when using tirzepatide alone or with other hypoglycemic drugs (Ludvik et al., 2021; Kadowaki et al., 2022).

#### 4.2.3 ADR of liver and gallbladder system

Jaundice and acute liver injury are examples of hepatobiliary adverse reactions. Tirzepatide is beneficial to the liver, resulting in a significant reduction in liver fat content (Gastaldelli et al., 2022). The specific impact mechanism is still unknown. However, in this study, the liver aminotransferase in four patients exceeded the normal upper limit by threefold, and two of these patients experienced fluctuations in aminotransferase levels during treatment. The author speculates that tirzepatide causes rapid fat mobilization and increased liver enzyme levels. Four out of five patients in a phase 2a study of a selective glucocorticoid receptor modulator for treating metabolic dysfunction-related fatty liver disease increased their liver enzyme levels by more than 250 IU/mL. It is speculated that changes in liver enzyme levels are linked to rapid liver delipidation (Kowdley et al., 2021).

In this study, two cases of liver damage occurred soon after the dose increase, one of which did not strictly adhere to the protocol of increasing the dose by 2.5 mg every 4 weeks. Rapid weight loss, acute dehydration, and malnutrition could have reduced functional liver reserves, resulting in tirzepatide-induced liver damage. The fluconazole used in the treatment may have impaired this patient’s liver function (Terrington et al., 2024). It is recommended that patients taking tirzepatide have their liver function closely monitored, particularly during dose changes and when combined with drugs that can cause liver damage.

#### 4.2.4 Nervous system ADR

Foot drop, paranoia, hallucination, and migraine are examples of nervous system adverse reactions. Foot sagging caused by common peroneal neuropathy is more common in studies involving rapid weight loss (Weyns et al., 2007; Şen et al., 2020), and its mechanism may be linked to the loss of fat pads that protect common peroneal nerves (Shields et al., 2021). Previous research has shown that the risk of common peroneal neuropathy is higher in those who lose weight quickly within 5–11 months (Weyns et al., 2007; Lale et al., 2020), which is highly consistent with the reported cases (7–8 months after use). In a multicenter study including 69 patients with peroneal mononeuropathy at the fibular head level, weight loss was the fourth most common etiology (14.5%), following posture (23.2%), surgery (21.7%), and unknown causes (16%), but ranking higher than trauma (10.1%) and external compression (5.8%) (Denys and Vrieze, 2023). For patients using rapid weight-loss medications, slow down the weight loss rate through close monitoring and dose adjustment to reduce the risk of complications like common peroneal neuropathy and foot drop. One patient in this study had headache hallucinations and a history of anxiety and depression but only developed paranoia and visual hallucinations after increasing the dose of tirzepatide, and the symptoms resolved after discontinuing the medication. The World Health Organization’s (WHO) global suspicious ADR database and alert database revealed suicide ideation signals associated with semaglutide (Schoretsanitis et al., 2024), and the risk ratio of suicidal ideation to semaglutide, liraglutide, and tirzepatide increased significantly (Mcintyre et al., 2025). It suggests that tirzepatide has a high risk of causing nervous system adverse reactions and should be monitored closely.

#### 4.2.5 Circulatory system ADR

The circulatory system’s ADR is hypotension, associated with or without tachycardia and venous thrombosis in the lower limbs. All three patients with blood pressure drops took GLP-1RA (semaglutide and duraglutide) but did not experience symptomatic hypotension. The experiments confirmed that the antihypertensive effect of 10 mg or 15 mg/week is greater than that of semaglutide 1 mg/week, and the antihypertensive effect of 5 mg, 10 mg, and 15 mg/week is dose-dependent (Dahl et al., 2022). The antihypertensive effect of tirzepatide may not be due to weight loss alone; a meta-analysis found that every 1 kg of weight loss reduced blood pressure by 1 mmHg (Drug Interaction Results Between Tirzepatide and Sacubitril/Valsartan M C, 2023). The blood pressure drop in one patient was consistent with this rule, but the drop in the other two cases far exceeded it (weight loss of 1 kg is approximately 2.9 mmHg and 10 mmHg). Tirzepatide’s mechanism for lowering blood pressure is multifactorial, and better monitoring of vital signs and capacity status is required.

Obesity and the risk of dehydration from decreased intake are common risk factors for venous thromboembolism (VTE) (Prandoni, 2005). The study found that participants who lost weight had a higher risk of VTE (Horvei et al., 2016). The study discovered that semaglutide increased the risk of VTE by 266% (relative risk was 3.66), raising concerns about its applicability among high-risk groups in VTE and reminding clinicians (Yin et al., 2021) to pay attention to the symptoms and signs of VTE that must be monitored during the treatment of GLP-1RA medications.

#### 4.2.6 Increased blood drug concentration causes ADR

In this study, three patients experienced ADRs due to elevated drug concentrations from polypharmacy. The reason could be that the combined drug (e.g., levothyroxine sodium) failed to adjust the dose based on weight loss, resulting in thyroid toxicity; delayed gastric emptying and other causes increased drug absorption (e.g.,6-mercaptopurine), causing liver damage; competing with the drug (e.g., sodium valproate) with plasma albumin, increasing thefree drug concentrations, triggering a toxic reaction. When combined with dosage-adjusted drugs based on body weight and drugs with a high plasma protein binding rate, it is critical to monitor drug concentration in real-time and adjust the dose accordingly.

### 4.3 Limitations

This study is limited by its small sample size and restriction to case reports, which may have omitted relevant data. Additionally, tirzepatide’s short marketing period results in less relevant data, with most case reports being newly published. The analysis cited additional GLP-1RA-related research reports. The results may be biased; Further real-world studiesare required to demonstrate the relationship between drugs and ADR and underlying mechanisms.

## 5 Conclusion

Tirzepatide is a novel therapy forglycemic control in adults and long-term weight management in obese/overweight patients. Its excellent glycemic reduction, weight loss effect, and low ADR incidence make it popular among doctors and patients. However, in clinical applications, it is critical to monitor ADRs, particularly those that are not listed in the instructions, such as ketoacidosis, foot drop, and lower limb venous thrombosis. Some ADRs, such as acute liver function damage and severe systemic allergic reactions, are mentioned in the instructions, but their severity is high. The clinical application requires enhanced therapeutic drug monitoring for patients with liver/renal insufficiency, underlying cardiovascular diseases, combined drug use with flexible-dose adjustment, and rapid weight loss. In the future, long-term and real-world research are required to validate these findings.

## Data Availability

The original contributions presented in the study are included in the article/Supplementary Material, further inquiries can be directed to the corresponding author.
