# Supplementary material for: A retrospective observational study on case reports of adverse drug reactions (ADRs) to tirzepatide
Source: Front Pharmacol. 2025 Jul 1;16:1608657. doi: 10.3389/fphar.2025.1608657 (PMC12259682; doi:10.3389/fphar.2025.1608657)
Supplement: Supplementary file 1 [file Table1.docx]

Table 1 Characteristics of included patients

| Country publication time | gender | age | Primary disease | Clinical manifestations | Dosage when ADR occurs | First time | Intervention measures | Better or not | Return time | Drug rechallenge | Combined medication | ADR correlation | ADR re-evaluation |
| --- | --- | --- | --- | --- | --- | --- | --- | --- | --- | --- | --- | --- | --- |
| United States 2025^[5]^ | female | 73 | Type 2 diabetes, hyperlipidemia, depression, obesity (BMI 38.5 kg/m2) | Sharp right lower abdomen, stinging aggravated for 3 days (severity is 9/10), nausea, vomiting, diarrhea, suggesting appendicitis | - | 1 week after medication | Piperacillin/tazobactam, rehydration, pain relief, post-appendectomy removal | yes | 1 week | no | Metformin, atorvastatin, omeprazole, bupropion | - | Probable |
| United States 2024^[6]^ | female | 66 | Recurrent abdominal wall incision hernia, history of multiple abdominal surgery, small intestinal obstruction, type 2 diabetes, obesity | Abdominal pain, nausea, vomiting, and hiccups for 2 days, indicating small intestinal obstruction | 2.5 mg/w | 2 weeks after medication | Stop the medicine, place the nasogastric tube and replenish the fluid | yes | - | no | - | - | Probable |
| United States 2023^[7]^ | male | 64 | History of diabetes, hypertension, hernia surgery, cholecystectomy and laparotomy | Nausea, vomiting, indigestion and excessive hiccups were seen on the second day of the first injection, followed by abdominal cramps, vomiting and watery diarrhea. Indicates intestinal obstruction. | - | first | Symptom-based treatment | yes | 6 days | - | - | - | Probable |
| United States 2023^[8]^ | male | 61 | Type 2 diabetes, hypertension and hyperlipidemia | Severe upper abdominal compression-like pain (7/10), suggesting small intestinal obstruction | 2.5mg/w to 5mg/w | 5 weeks after medication | Receive conservative treatment | yes | - | no | - | - | Probable |
| United States 2023^[9]^ | male | 65 | Obesity (BMI 40.7 kg/m2) and type 2 diabetes, | Watery diarrhea and pain in the upper right abdomen. CT shows intestinal ischemia and thickening of the stomach wall, indicating intestinal ischemia | - | One week after medication | Treatment and antibiotic therapy | yes | - | no | - | - | Possible |
| United States 2024^[10]^ | female | 62 | Obesity (BMI40 kg/m2), venous thromboembolism, heart failure, benign polyps, diverticulum disease | Acute abdominal pain and bloody stool last for 1 day, and the intestinal peristalsis slows down, indicating colonic ischemia | 2.5 mg/w | 4 weeks after medication | Intravenous infusion, intestinal rest, antibacterial treatment, pain relief | yes | 1 week | - | apixaban, spironolactone, furosemide, atorvastatin, fluoxetine, lisinopril, atenolol | - | Possible |
| Nigeria 2023^[11]^ | male | 58 | Type 2 diabetes | Repeated bloody stools, 3-4 diarrhea, liver mass, and extra fluid near the spleen and liver, indicating stage IV pancreatic cancer. | - | Use medicine for 9 months | Chemotherapy | no | - | no | - | - | Possible |
| United States 2024^[12]^ | male | 59 | Type 2 diabetes, hyperlipidemia, hypertension | Acute upper abdominal pain, vomiting, leukocytosis, tachycardia, imaging support pancreatitis | 7.5mg/w | Use medicine for 2 days | Analgesics, antibiotic treatment, support measures | yes | - | no | - | - | Probable |
| United States 2023^[13]^ | female | 27 | Weight loss | Severe abdominal pain, nausea, bile vomiting and watery diarrhea, suggesting high-level colon obstruction | - | Four months later, the dose was increased just before the onset of the disease | Anti-infection and symptomatic supportive treatment, colonoscopy failed to successfully remove fecal impaction. Final total abdominal colectomy. | yes | - | no | - | - | Probable |
| United States 2024^[14]^ | female | 64 | Hypertension, hyperlipidemia, obesity | Sudden upper abdominal pain, acute hypoxic respiratory failure, and the count of lipase, blood sugar, hemoglobin, and white blood cell were higher than normal. Severe necrotizing pancreatitis. | - | - | Symptom-based supportive treatment, infusion of red blood cells and platelets, anti-infection, continuous renal replacement therapy. Eventually die. | no | none |  | - | - | Possible |
| United States 2023^[15]^ | female | 38 | Prediabetes, anxiety, major depression | Watery diarrhea, nausea and vomiting persistent and severe pain (intensity of 10/10), radiated to the bilateral back and worsened after eating. Slight pancreatitis | 7.5mg/w | 2 months after medication | Symptom-based treatment | yes | - | no | Escitalopram, esomeprazole, loperamide, ketoolic acid | - | Possible |
| United States 2023^[16]^ | female | 59 | Uterine polyps, obesity | Hysteroscopic polypsy, the oropharynx of gastric content reflux, suggesting delayed gastric emptying | - | - | Put the gastric tube into the mouth and aspirate 500 ml of viscous stomach contents | yes | - | - | - | - | Possible |
| United States 2025^[17]^ | male | 57 | Type 2 diabetes, hypertension | Right abdominal cramps (8/10), non-hemorrhagic vomiting after eating, intermittent diarrhea, dehydration, and sunken eyes. Suggesting gastric outlet obstruction | 2.5 mg/w | Metformin was converted to this drug shortly after, not mentioned | Intestinal decompression, infusion of Lactate Ringer | yes | - | no | Glipizine, losartan, tadalafil | - | Probable |
| United States 2024^[18]^ | male | 46 | Stenosis Crohn's disease urocolitis disease | Progressive nausea, vomiting and abdominal pain, gastric plant stones extending to cardia, and gastric dilation | - | 1 week after medication | Pain, chemically dissolved stones are not effective, esophageal gastroduodenoscopy removes gastric stones | yes | - | - | - | - | Probable |
| Kuwait 2024^[19]^ | female | twenty four | Obesity (BMI34 kg/m2) | Repeated vomiting, nausea and abdominal pain, which eventually showed acute hepatitis and impaired coagulation function | 2.5 mg/w to 10mg/w, last time 12 mg | 5 months after medication | Intravenous infusion, stomach protection, antiemetic, and plasma infusion, vitamin K injection, lactulose, rifaximin and ursodeoxycholic acid | yes | 10 days | - | Oral contraceptives | Possible | Probable |
| United States 2024^[20]^ | female | 42 | Inflammatory bowel disease, obesity (BMI31 kg/m2), hypercholesterolemia | AST44 U/L, ALT75 U/L. Increased concentration of 6-TGN and 6-MP drugs, indicating liver injury | 2.5mg/minimum to 7.5mg/w | 4 months after medication | No stopping the medication, reduce the dose of 6-MP 75 mg to 50 mg | yes | 6 weeks | yes | 6-mercaptopurine | - | Probable |
| United States 2024^[21]^ | female | 37 | Obesity, hypercholesterolemia, hypertension | ALT500 U/L, AST261 U/L, suggests liver injury | - | 2.5 months after medication | Stop the medicine | yes | 3 weeks | Yes, after three weeks, the aminotransferase increased again and returned to normal two months after stopping the drug. | levonorgestrel | Probable | Probable |
| United States 2024^[22]^ | male | 64 | Hypertension, hyperlipidemia, type diabetes, obesity (BMI 30 kg/m2) | Nausea, vomiting and epigastric pain for 1 week, accompanied by AST 629 IU/L, ALT 1093 IU/L, ALP 176 IU/L, total bilirubin 5.8 mg/dL, suggesting jaundice | 2.5 mg/w | 6 weeks after medication | Discontinue medication treatment | yes | 6 weeks | - | Ramipril, hydrochlorothiazide, rosuvastatin | Highly possible（Unspecified） | Possible |
| UK 2024^[23]^ | female | 33 | - | Acute vomiting with chest and abdominal pain for 3 weeks, hyponatremia, and dehydration signs. CT showed emphysema mediastinum with increased neck emphysema, and esophageal perforation, indicating acute liver failure | 5mg/w to 10mg/w | 4.5 months after medication | Intravenous potassium supplementation, piperacillin/tazobactam and fluconazole, acetylcysteine ​​and vitamin K | yes | 2 weeks | - | - | Possible | Probable |
| Kuwait 2024^[24]^ | female | twenty one | Obesity (BMI28.2 kg/m2) | Abdominal pain, diarrhea (3 to 4 times per day) vomiting, suggesting ketoacidosis and normal blood sugar levels | 5mg/w | Use medication for 3 weeks | Intravenous fluid replenishment, oral ondansetron, intravenous drip pantoprazole | yes | 4weeks | - | none | Probable | Probable |
| United States 2025^[25]^ | male | 35 | Type 2 diabetes (not insulin used), hypertension, hyperlipidemia, hypogonadism, BMI20.7 kg/m² | Tenderness, vomiting (0.5-1 hour/time), chills, reflux, presence of urinary ketones, blood ketones, fatigue, tachycardia, mild dyspnea, suggesting ketoacidosis and normal blood sugar levels | 2.5 mg/w | - | Replenish fluid, antiemetic, and intravenous potassium supplement | yes | 2 days | - | Empalifen | - | Possible |
| Kuwait 2024^[26]^ | female | 29 | Obesity (BMI32 kg/m2), fatty liver level 1 | After the dose increased to 5 mg, abdominal pain (5 days) and vomiting (3 times/day, 5 days), indicating hypoglycemia ketoacidosis | 2.5 mg/w (4weeks) to 5 mg/w (3weeks) | 7 weeks after medication | Intravenous lactate Ringer solution | yes | 4 weeks | no | Unspecified | - | Probable |
|  | female | 43 | Obesity (BMI31.3 kg/m2), fatty liver level 1 | After the dose increased to 5 mg, abdominal pain (7 days), vomiting (4 times/day, 2 days) and diarrhea (3 days), indicating hypoglycemia ketoacidosis | 2.5 mg/w (4weeks) to 5 mg/w (2weeks) | 6 weeks after medication | Intravenous fluid | yes | 4 weeks | Yes (low dose) | Unspecified | - | Probable |
|  | female | 17 | Obesity (BMI=30.4 kg/m2), fatty liver level 1 | The 5th week of abdominal pain (2 days), vomiting (3 times/day, 2 days) and diarrhea (3 days), indicating hypoglycemia ketoacidosis | 2.5 mg/w (4weeks) to 5 mg/w(1 week) | 5 weeks after medication | Intravenous fluid | yes | 4 weeks | Unspecified | Unspecified | - | Probable |
|  | female | 26 | Obesity (BMI30.8 kg/m2), fatty liver level 1 | Abdominal pain (4 days), frequent exercise relaxation and vomiting (3 times/day, 1 day), diarrhea (3 days), suggesting hypoglycemia ketoacidosis | 2.5 mg/w (4weeks) to 5 mg/w (2weeks) | 6 weeks after medication | Intravenous fluid | yes | 4 weeks | Unspecified | Unspecified | - | Probable |
| United States 2024^[27]^ | male | 72 | Type 2 diabetes, hyperlipidemia and obesity | Nausea, vomiting and abdominal pain, suggesting normal blood sugar diabetic ketoacidosis | - | 2 days after taking the medicine | Replenish fluids and replenish electrolytes | yes | - | no | Engaliflozin and metformin | - | Probable |
| United States 2023^[28]^ | female | 29 | Overweight (BMI 26.5 kg/m2) | Severe gastrointestinal adverse reactions, leading to starvation ketoacidosis | 2.5 mg/w | 19 days after medication | Replenish fluid, antiemetic, and replenish electrolytes | yes | - | - | - | - | Probable |
| United States 2024^[29]^ | male | 62 | Obesity (BMI31.2 kg/m2), hypothyroidism, type 1 diabetes | Palpitations, sweating and confusion in consciousness, fever, and tremor in hands, indicating thyroid toxicity and atrial fibrillation | 2.5mg/w increase to 10mg/w | 6 months after medication | - | yes | - | - | Levothyroxine, multiple times a day insulin | - | Probable |
| United States 2023^[30]^ | female | 26 | BMI26.5 kg/m2 | Nausea, vomiting, suggesting ketoacidosis (the blood sugar level is not known or the normal level) | 2.5mg/w | 3 weeks after medication | Antiemetic, intravenous fluid and electrolytes | yes | 3 days | no | none | - | Probable |
| United States 2024^[31]^ | female | 67 | Type 2 diabetes, obesity, hypercholesterolemia, hypertension | Generalized urticaria with severe itching, spreading throughout the body, excluding the face and neck, indicating generalized allergies | - | After about 10-15 minutes of administration | Anti-allergic treatment | yes | - | no | Insulin glargine, metformin | - | Probable |
| United States 2023^[32]^ | male | 67 | Hypertension, hyperlipidemia, type 2 diabetes, obstructive sleep apnea | Diffuse urticaria, swelling of the throat, shortness of breath, wheezing and dizziness, diarrhea, and incontinence of the stool, suggesting allergies, bipolar allergic reactions | 5mg/w | About 20 minutes later | Intramuscular injection of epinephrine, intramuscular injection of methylprednisolone and diphenhydramine. After the improvement, the symptoms reappear, and intramuscular injection of epinephrine was given. Post-transfer to the intensive care unit | yes | 1 day | no | - | - | Probable |
| Japan 2023^[33]^ | male | 76 | Obesity, type 2 diabetes and alcoholic cirrhosis. | The first injection showed rash and pain at the injection site. It gradually expanded in the following days with dull pain and burning. After 10 days, swelling and rash cover the entire lower abdomen, indicating allergies and pain | 2.5 mg/w | First injection | Disable | yes | One month | - | Engaliflozin, glimeride | - | Probable |
| United States 2024^[34]^ | male | 77 | Obesity (37.51 kg/m2), pre-diabetes, coronary artery disease, hyperlipidemia, stage 3a chronic kidney disease, chronic compensatory heart failure with reduced ejection fraction, lumbar decompression, and history of left total knee arthroplasty | Both feet drooping, decreased muscle strength on both sides of the ankle joint, light sense of touch on the lateral side of the left calf, acute exacerbation of chronic common peroneal neuropathy in the small head of the bilateral fibula (more serious on the right), no evidence of lumbar nerve radiculopathy | - | 7 months after medication | Surgery is released and received orthotic treatment and physical rehabilitation. | yes | - | - | - | - | Probable |
|  | female | 56 | History of type 2 diabetes, depression, anxiety, systemic lupus erythematosus, Sjogren's syndrome, attention deficit hyperactivity disorder | The right foot is drooping, the muscle strength of the anterior muscle/extensor longus is decreased, and the muscle strength of the remaining lower limbs is normal. After April, the left foot sags | 2.5mg/minimum to 15mg/w | 8 months after medication | Bilateral peroneal nerve release | yes | - | - | Metformin | - | Probable |
| United States 2024^[35]^ | female | 43 | Obesity (BMI32 kg/m2) | Hypoglycemia, persistent migraine | - | - | Coenzyme q10, D-ribose, vitamin B complex and acetyl-L-carnitine | yes | - | - | - | - | Probable |
| United States 2024^[36]^ | female | 61 | History of anxiety and depression, overweight, but no weight-related comorbidities | Headache, vomiting, diarrhea, paranoia and visual hallucinations | - | After the dose increases, no specific mention | Stop the medicine | yes | 8 days | - | - | - | Probable |
| United States 2023^[37]^ | male | 50 | History of epilepsy, weight loss | Anorexia worsens and worsens into extreme fatigue, ataxia and encephalopathy, and increases in sodium valproate concentration | 2.5 mg/w | 3 weeks after medication | Discontinue sodium valproate and treat levocarnitine | yes | 6 days | - | Sodium valproate sustained release tablets, levetiracetam | - | Possible |
| United States 2024^[38]^ | male | 63 | Non-ischemic cardiomyopathy, cardiac function level II, heart failure, hypertension, hypothyroidism, obstructive sleep apnea, diverticulitis (BMI 45.8kg/m2) | Hypotension | 2.5 mg/minimum 12.5 mg/weekly 2.5 mg | 4 months after medication | Hypotension, heart rate increases, carvedilol dose is halved, syncope attacks and hypotension 6 months later, sacubali/valsartan is reduced to halves, carvedilol is halved again | yes | One month | - | Sacubatre/valsartan, carvedilol, spironolactone | Probable | Probable |
|  | male | 64 | Ischemic cardiomyopathy, cardiac function level II, heart failure, type 2 diabetes, coronary artery bypass surgery, hypertension, obesity (BMI 38.3 kg/m2), sleep apnea | Dizziness, heart failure, hypotension, heart rate increase, serum creatinine and urea nitrogen increase | 10mg/wto 12.5mg/w | 5 months after medication | Torasemide dose is reduced until the drug is stopped, and the dose of sakubaprectin/valsartan is reduced by half | yes | - | - | Sacubali/valsartan, metoprolol succinate, torasemide | Probable | Probable |
|  | male | 69 | History of ischemic cardiomyopathy cardiac function grade II, heart failure, type 2 diabetes, 2 coronary artery bypass transplantation | Dizziness, hypotension, and heart rate increase | 7.5mg/w | 2 months after medication | After changing sakubali/valsartan to valsartan, blood pressure is still low. Reduce the dose of valsartan, and blood pressure is restored after one week, and the dose of valsartan is restored. | yes | 1 week | - | Metoprolol succinate, sacubatre/valsartan, engaliflozin | Probable | Probable |
| UAE 2024^[39]^ | male | 20 | Obesity (BMI>35kg/m2), glucose-6-phosphate dehydrogenase deficiency, childhood asthma | Pain and swelling of the leftleg for 3 days (7/10 minutes), mild tenderness and mild redness, and the left thigh and calves are thicker than the right leg, indicating extensive venous thrombosis of the lower limbs | 7.5mg/w | - | Alteplase thrombolysis, mechanical thrombectomy and anticoagulation | yes | - | - | - | - | Possible |
| United States 2023^[40]^ | female | 42 | Hypertension, diabetes | Acute and rapid decline of GFR and increased blood urea nitrogen suggest acute renal injury | - | - | Intravenous fluid replenishment, potassium supplementation, etc. | yes | 2 days | - | Losartan/hydrochlorothiazide, amlodipine, labelor | - | Probable |
| United States 2025^[41]^ | female | 46 | Weight loss | Itchy brown plaques on both sides of the armpit and groin, accompanied by a small amount of scales, suggesting pigmented lichen planus | - | 6-7 months | Tacrolimus ointment | yes | - | yes | - | - | Possible |
